# Development and validation of the Salzburg COPD-screening questionnaire (SCSQ): a questionnaire development and validation study

**DOI:** 10.1038/s41533-016-0005-7

**Published:** 2017-01-26

**Authors:** Gertraud Weiss, Ina Steinacher, Bernd Lamprecht, Bernhard Kaiser, Romana Mikes, Lea Sator, Sylvia Hartl, Helga Wagner, M. Studnicka

**Affiliations:** 1 Department of Pneumology, Paracelsus Medical University, Salzburg, Austria; 2 Department of Pulmonary Medicine, Kepler-University-Hospital, Linz, Austria; 3 Faculty of Medicine, Johannes-Kepler-University, Linz, Austria; 4 Ludwig Boltzmann Institute of COPD and Respiratory Epidemiology, Otto Wagner Hospital, Vienna, Austria; 5 Department for Statistics, University of Linz, Linz, Austria

## Abstract

Chronic obstructive pulmonary disease prevalence rates are still high. However, the majority of subjects are not diagnosed. Strategies have to be implemented to overcome the problem of under-diagnosis. Questionnaires could be used to pre-select subjects for spirometry and thereby help reducing under-diagnosis. We report a brief, simple, self-administrable and validated chronic obstructive pulmonary disease questionnaire to increase the pre-test probability for chronic obstructive pulmonary disease diagnosis in subjects undergoing confirmatory spirometry. In 2005, we completed the Austrian Burden of Obstructive Lung Disease-study in 1258 subjects aged >40 years. Post-bronchodilator spirometry was performed, and non-reversible airflow limitation defined by FEV_1_/FVC ratio below the lower limit of normal. Questions from the Salzburg chronic obstructive pulmonary disease screening-questionnaire were selected using a logistic regression model, and risk scores were based on regression-coefficients. A training sub-sample (*n* = 800) was used to create the score, and a test sub-sample (*n* = 458) was used to test it. In 2008, an external validation study was done, using the same protocol in 775 patients from primary care. The Salzburg chronic obstructive pulmonary disease screening questionnaire was composed of items related to “breathing problems”, “wheeze”, “cough”, “limitation of physical activity”, and “smoking”. At the >=2 points cut-off of the Salzburg chronic obstructive pulmonary disease screening questionnaire, sensitivity was 69.1% [95%CI: 56.6%; 79.5%], specificity 60.0% [95%CI: 54.9%; 64.9%], the positive predictive value 23.2% [95%CI: 17.7%; 29.7%] and the negative predictive value 91.8% [95%CI: 87.5%; 95.7%] to detect post bronchodilator airflow limitation. The external validation study in primary care confirmed these findings. The Salzburg chronic obstructive pulmonary disease screening questionnaire was derived from the highly standardized Burden of Obstructive Lung Disease study. This validated and easy to use questionnaire can help to increase the efficiency of chronic obstructive pulmonary disease case-finding.

## Chronic obstructive pulmonary disease: Questionnaire for pre-screening potential sufferers

Scientists in Austria have developed a brief, simple questionnaire to identify patients likely to have early-stage chronic lung disease. Chronic obstructive pulmonary disease (COPD) is notoriously difficult to diagnose, and the condition often causes irreversible lung damage before it is identified. Finding a simple, cost-effective method of pre-screening patients with suspected early-stage COPD could potentially improve treatment responses and limit the burden of extensive lung function (‘spirometry’) tests on health services. Gertraud Weiss at Paracelsus Medical University, Austria, and co-workers have developed and validated an easy-to-use, self-administered questionnaire that could prove effective for pre-screening patients. The team trialed the five-point Salzburg COPD-screening questionnaire on 1258 patients. Patients scoring 2 points or above on the questionnaire underwent spirometry tests. The questionnaire seems to provide a sensitive and cost-effective way of pre-selecting patients for spirometry referral.

## Introduction

Chronic obstructive pulmonary disease (COPD) is the third leading cause of death globally in 2010.^1^ Nevertheless, available morbidity data greatly underestimate the burden of COPD.^2–4^ Due to the insidious nature of COPD, the disease usually progresses unnoticed in many subjects and causes irreversible lung damage. Therefore, early diagnosis of COPD is crucial. The results of the international Burden of Obstructive Lung Disease study (BOLD) have shown that the majority of subjects with post bronchodilator (PBD) FEV_1_/FVC< lower limit of normal (LLN) are not diagnosed.^3–5^ Although many of those undiagnosed have mild disease, they are the group with the greatest potential health gain from timely intervention and treatment.

The risk for COPD increases with age and cumulative exposure to inhalational injury, mainly tobacco smoking.^7^ Therefore, smoking cessation is the most effective way to reduce further loss of lung function.^8^ In the Lung Health Study, smoking intervention significantly reduced the decline of FEV_1_ in smokers, aged 35 to 60 years with mild-to-moderate COPD.^9^ Furthermore, knowledge about one’s abnormal lung function has been shown to be positively associated with successful smoking cessation in some studies^10,11^ while others have not corroborated this finding.^12,13^ Early pharmacological treatment of mild to moderate COPD increases lung function improves quality of life and prevents exacerbations.^14^ The TORCH and UPLIFT trials have demonstrated these benefits in COPD patients with FEV_1_% predicted below 60% and 70%, respectively.^15,16^

Given the impact and the natural history of COPD as well as the available treatment options, subjects with COPD should be identified early. While non-selective spirometry screening is no longer recommended^17^ strategies combining questionnaire and spirometry are considered a promising tool for early detection.^18,19^

A number of COPD case finding-questionnaires have been reported previously^20–26^ All of these tools use similar items related to respiratory symptoms like wheeze, dyspnea, and sputum production. However, these studies were conducted in different settings and populations. Some studies restricted the analysis to smokers, while others to selected settings (i.e., primary care offices).^21–23,25–30^ Another possible limitation of these studies is the method of diagnosing COPD. Either, COPD diagnosis was based on pre-bronchodilator spirometry only^20,24^ or COPD was diagnosed using the fixed ratio (FEV_1_/FVC < 0.70) rather than the lower limit of normal (FEV_1_/FVC < LLN)^21–25^ in either case resulting into overdiagnosed COPD. Additionally, some of the questionnaires were not validated at all or failed to show external validity.^22–32^

We herein report the Salzburg COPD screening questionnaire (SCSQ), which is based on the highly standardized international BOLD protocol including PBD spirometry, and validated in a random sample of primary care patients.^33^ It was our aim to develop a brief, simple, and easy to use COPD questionnaire to pre-select subjects for spirometry.

## Results

### Development of the SCSQ in the population-based BOLD-sample

**Fig. 1:**
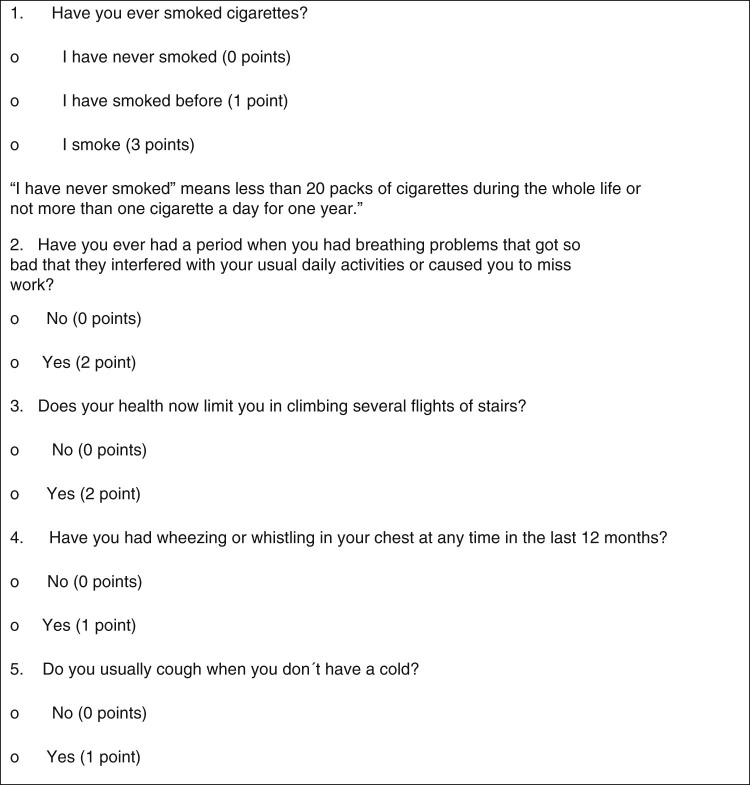
Items of the Salzburg COPD–screening questionnaire (SCSQ)

Altogether, 1258 subjects from the Salzburg BOLD study with complete spirometry and questionnaire data were included. Information on participating subjects can be found elsewhere.^5^ When the training and testing sub-sample were compared, no significant difference was detected regarding population characteristics (Table 1). Age, sex, education, respiratory symptoms, smoking and perception of health were significantly associated with non-reversible PBD FEV_1_/FVC < LLN. In subsequent multivariate analysis, the following items had the greatest impact on the scoring model (score of each question is in parentheses): “current smoking” (3 points), “ex-smoking” (1 point), a reported “period of breathing problems that interfered with daily activities or make you unable to work” (2 points), and “shortness of breath limiting in climbing several flights of stairs” (2 points). Regression coefficients, corresponding odds ratios, and scoring points are reported in Table 2. Inclusion of interaction terms did not significantly improve the model. The final COPD risk questionnaire is shown in Fig. 1.

**Table 1:** Comparison of the training and test sub-sample

	Training sub-sample (*N* = 800)	Test sub-sample (*N* = 458)	*p*-value
Age in years (mean, SE)	57.6 (0.40)	57.9 (0.53)	0.680
Male sex (%)	430 (53.8%)	255 (55.7%)	0.509
Years of education > 12	120 (15.0%)	67 (14.6%)	0.859
Risk-Factors			
Smoking status			
Never smoker	368 (46.0%)	227 (49.6%)	0.207
Former smoker	282 (35.3%)	139 (30.4%)	
Current smoker	150 (18.8%)	92 (20.1%)	
Age in years at start smoking (mean, SE)	18.9 (0.26)	18.7 (0.34)	0.472
Pack-years smoking > 20	202 (25.3%)	121 (26.4%)	0.176
Worked in a dusty job	227 (28.4%)	119 (26.0%)	0.361
Farming	187 (23.4%)	101 (22.1%)	0.591
Passive smoking at home	178 (22.3%)	100 (21.8%)	0.864
Symptoms			
Cough without a cold	131 (16.4%)	92 (20.1%)	0.100
Phlegm	191 (23.9%)	121 (26.4%)	0.315
Wheezing or whistling at any time in the last 12 months	99 (12.4%)	66 (14.4%)	0.804
Shortness of breath interfering with daily activity	88 (11.0%)	36 (7.9%)	0.072
Own view about health “excellent”	90 (11.3%)	55 (12.0%)	0.685
Impact			
Breathing problems interfered with activity or caused to miss work	14 (1.8%)	8 (1.8%)	0.999
Health limits climbing several flights of stairs	184 (23.0%)	94 (20.5%)	0.309

*p*-value (*p* < 0.05) refer to comparison of training and test subsample

**Table 2:** Multivariate predictors of PBD FEV1/FVC <LLN1- results from the population-based training subsample (*n* = 800)

		Coefficients	Points	Odds ratio (95% CI)	*p*-value
Have you ever smoked cigarettes?	Never	–	0	1 (–)	–
		1.26	3	3.53 (2.11;5.91)	<0.001
	Former	0.53	1	1.70 (1.06;2.73)	0.005
Breathing problems interfered with activity or make you unable to work?	Yes	0.75	2	2.11 (1.21;3.68)	0.0082
	No	–	0	1 (–)	–
Health limits in climbing several flights of stairs	Yes	0.83	2	2.29 (1.49;3.52)	<0.001
	No	–	0	1 (–)	–
Wheezing or whistling at any time in the last 12 months	Yes	0.54	1	1.71 (1.01;2.89)	0.044
	No	–	0	1 (–)	–
Cough without a cold	Yes	0.49	1	1.64 (1.0;2.67)	0.046
	No	–	0	1 (–)	–

Post-bronchodilator FEV1/FVC ratio below the lower limit of normal (LLN)

**Fig. 2:**
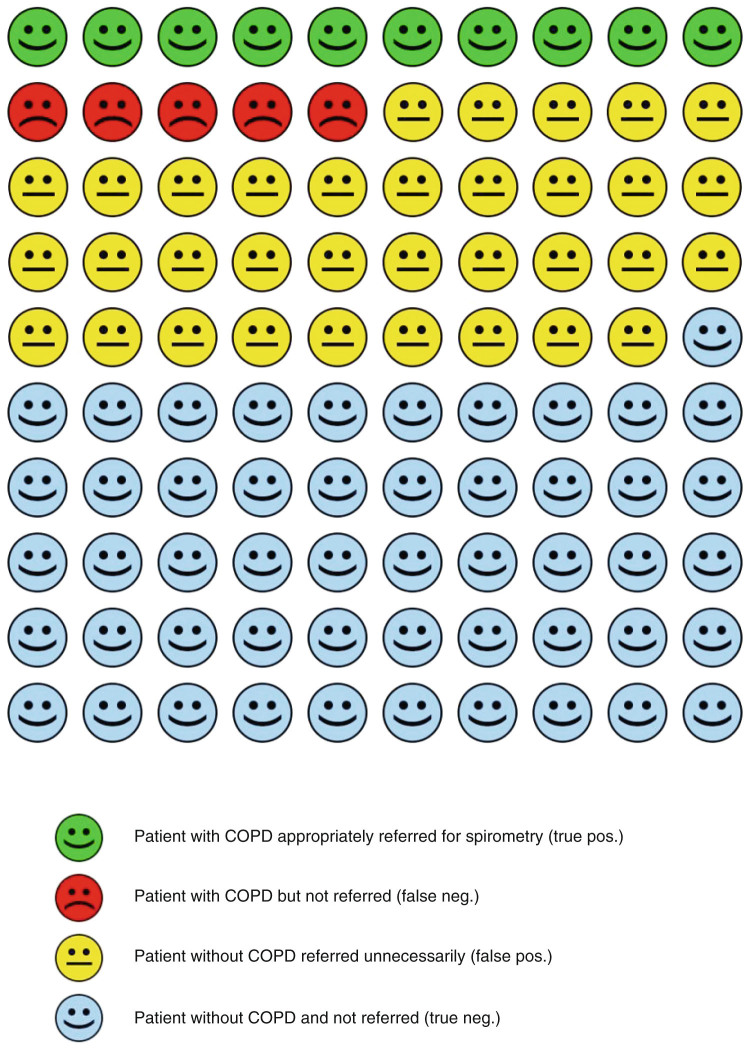
Diagnostic accuracy of the SCSQ in a hypothetical sample of 100 participants, 15 of whom have undiagnosed COPD

In the test subsample, 8.2% of subjects with a score of <2 points were found to have PBD FEV_1_/FVC < LLN. Therefore, a cut-off point ≥2 points was chosen to achieve maximum NPV to reliably exclude individuals unlikely to suffer from non-reversible airflow limitation. At this ≥2 point cut-off, sensitivity and specificity for non-reversible airflow limitation was 69.1% [95%CI: 56.6%; 79.5%], and 60.0% [95%CI: 54.9%; 64.9%] respectively, while the PPV was 23.2% [95%CI: 17.7%; 29.7%] and the NPV 91.8% [95%CI: 87.5%; 95.7%] (Fig. 2). At the ≥2 cut-off, the number needed to screen was 10 [95% CI: 7.4; 12.1]. Consequently, for 203 subjects who achieved SCSQ score ≥2 would get spirometry and 47 of those would be found obstructive. 89.4% (42/47) of these are symptomatic. At a ≥5 point cut-off, the risk-score had 27.9% sensitivity, 92.8% specificity, a PPV of 40.4% and a NPV of 88.1% (Table 3).

**Table 3:** Sensitivity, specificity, PPV, NPV, NNS of the SCSQ to predict PBD FEV1/FVC <LLN at different cutoff points as observed in the BOLD test sub-sample

	Cutoff point				
	≥1	≥2	≥3	≥4	≥5
Sensitivity	88.2%	69.1%	55.9%	41.2%	27.9%
Specificity	34.4%	60.0%	74.95%	86.7%	92.8%
PPV	19.0%	23.2%	27.9%	35.0%	40.4%
NPV	94.4%	91.8%	90.7%	89.4%	88.1%
NNS	8	10	12	16	24

*BOLD* Burden of Obstructive Lung Disease, *NNS* number needed to screen, *NPV* negative predictive value, PBD *FEV1/FVC <LLN* post-bronchodilator FEV1/FVC ratio below the lower limit of normal (LLN), *PPV* positive predictive value, *SCSQ* Salzburg COPD screening questionnaire

**Fig. 3:**
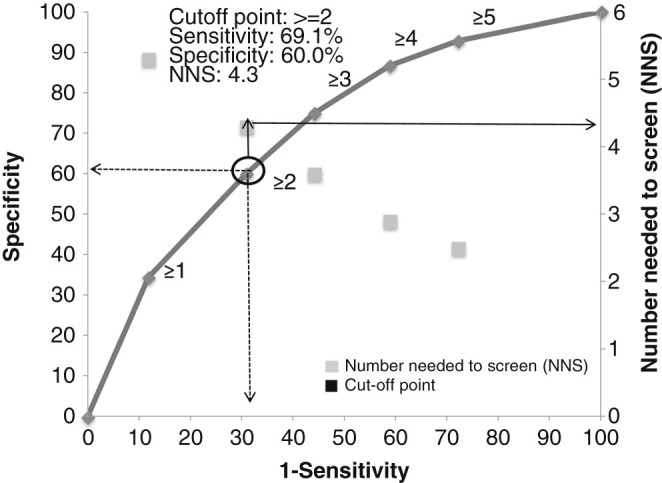
AUC analysis for different cutoff-points of the SCSQ for the population-based BOLD sample

Given the 15.8% prevalence of airflow limitation in the BOLD study, on average 6.5 subjects would need spirometry to detect one subject with PBD FEV_1_/FVC < LLN, and pre-screening with the SCSQ will result in a 33.8% reduction of this effort, because for a score ≥2, only 4.3 will need spirometries to detect a new subject with COPD. At a cut-off point of ≥2, 10 (9.7) subjects will need to complete the SCSQ to detect one COPD case (Table 3). Using a cut-off point of ≥5, the mean number of subjects to screen would be reduced to 2.5. We decided to use a ≥2 point cut-off, because it shows comparable sensitivity and specificity across age categories. The accuracy of the scoring model was characterized by an AUC of 0.71 (Fig. 3).

### Validation of the SCSQ in patients from primary care

A total of 775 patients of primary care offices completed the BOLD questionnaire and PBD spirometry met pre-set quality criteria.^33^ Fifty percent of the invited primary care offices were willing to participate in this study. The patients’ mean participation rate in the offices was 12.5%. Detailed information on characteristics of this study sample can be found elsewhere.^33^ In the primary care validation sample, prevalence of PBD FEV_1_/FVC < LLN was 9.8%. Population characteristics (age, gender, smoking status and education) were not significantly different between the BOLD and the primary care based validation sample (data not shown).^33^

For the primary care validation sample, the sensitivity at the cutoff point of ≥2 was 67.1% [95%CI: 55.3%; 77.2%], specificity 58.9% [95%CI: 55.2%; 62.6%], the PPV 15.1% [95%CI: 11.5%; 19.5%] and NPV 94.3% [95%CI: 91.6%; 96.2%], while for the cutoff point ≥5, sensitivity was 22.4%, specificity 93.4%, PPV 27.0% and NPV 91.7%.

**Fig. 4:**
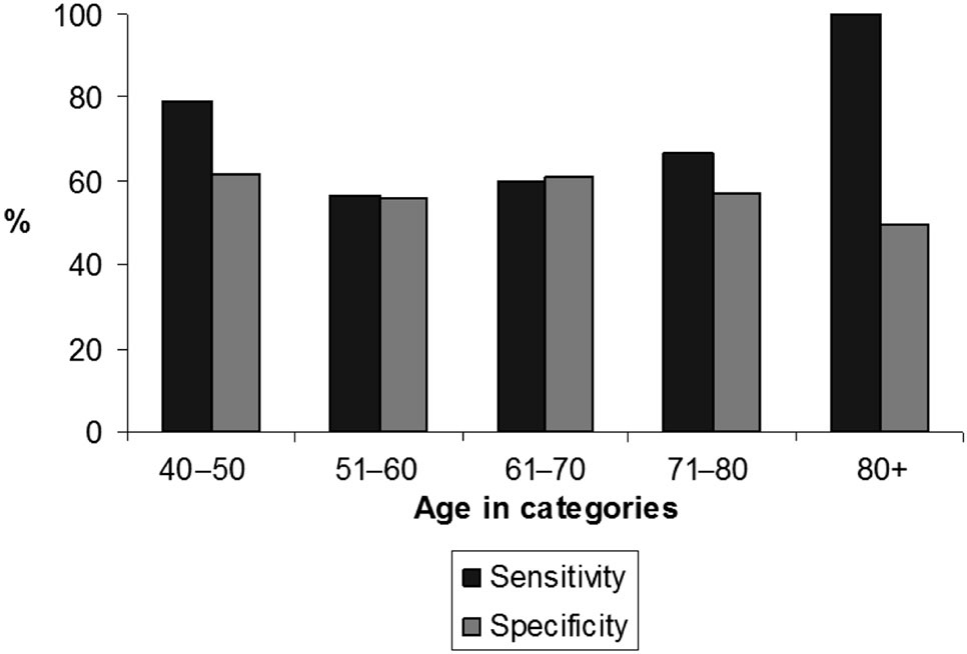
Age-specific sensitivity and specificity of the SCSQ (cut-off point >=2) to predict PBD FEV_1_/FVC <LLN in the primary care sample

When using the ≥2 point cutoff, the number of spirometries needed to detect a new case was 6.6, which represented a 35.3% reduction of the effort. The ≥2 point cut-off value also showed comparable sensitivity and specificity across age categories (Fig. 3). The AUC characterizing the accuracy of the scoring model in the primary care sample was 0.66 (Fig. 4).

## Discussion

### Main findings

We report our findings on screening for non-reversible airflow limitation. COPD is greatly underdiagnosed^2–4^ and the clinical diagnosis of COPD requires spirometry to confirm non-reversible airflow limitation.^17^ However, non-selected population screening with spirometry is expensive and no longer recommended. Therefore, using the validated SCSQ before spirometry may be a more reasonable and cost-effective approach.

We have developed and validated a simple COPD risk-score based on the highly standardized procedures of the BOLD study. Our scoring model was developed using population-based data and PBD spirometry. We demonstrate that the diagnostic effort to detect COPD by spirometry could be reduced when using a simple five-item-questionnaire. The SCSQ provides reasonable sensitivity for use as a tool to identify individuals with non-reversible airflow limitiation. The questions of the SCSQ are self-administrable and quick and easy to answer and can therefore be used for COPD screening in newspapers or the internet.^37^

### Interpretation of findings in relation to previously published work

Calverley et al. report an analysis using the population-based NHANES III-dataset to determine the most appropriate questions to diagnose COPD. The fixed ratio of FEV_1_/FVC < 0.7 was chosen to define airways obstruction, and the best performing variables for predicting FEV_1_/FVC < 0.7 were “age”, “smoking status”, “pack-years”, “wheeze”, “phlegm”, “body mass index“, and a “self reported diagnosis of chronic bronchitis, emphysema or asthma”. Eighty five percent sensitivity and 45% specificity, a PPV of 38%, and a NPV of 88% was seen.^20^ This analysis was restricted to smokers and did not use PBD lung-function. Given that COPD affects never smokers in 25–45% of cases^38,39^ we decided to include never smokers in our analysis.

Price et al. developed a COPD score to identify PBD FEV_1_/FVC < 0.7 in current or former smokers. This questionnaire was aimed to improve the efficiency of COPD diagnosis in primary care and included items related to age, smoking, and symptoms. A sensitivity of 80.4% and specificity of 72.0% were reported.^18,21,22^ This COPD score was validated externally in several studies, however findings were somehow inconsistent. Two studies reported this score is useful for the identification of subjects with airways obstruction^31,40^ while another study in the primary care setting observed that the score cannot discriminate between subjects with or without airways obstruction.^32^

Martinez et al. reported that age, breathlessness, productive cough, limitation of activity and smoking history predicted PBD airflow limitation.^23^ Patients aged 35 years and older were recruited at GP-offices. For the purposes of this study, airflow limitation was defined as PBD FEV_1_/FVC < 70. This COPD-Population-Screener Questionnaire (COPD-PS)^23^ was associated with a sensitivity of 84.4% and a specificity of 86.7%, PPV of 56.8% and NPV of 86.4%, and the AUC for the total score was 0.81. However, development of this questionnaire was based on interviews with COPD patients, rather than relying on validated questionnaires from epidemiological studies. The external validation of the COPD-PS was tested in a single population-based study and indicated limited generalizability of results.^41^

The Lung Function Questionnaire (LFQ) was developed using NHANES III data.^24^ The strengths of this five items questionnaire are its origin from a population-based data set and the adequate accuracy, sensitivity and specificity. In a primary care validation sample Hanania et al.^41^ confirmed the screening accuracy of the LFQ. However, the main limitation of the LFQ relate to pre-BD airflow limitation and use of the fixed ratio.

### Strengths and limitation of the SCSQ

As shown above, a number of COPD risk scores have been developed. However, most COPD samples were selected rather than population-based, did use pre-BD spirometry or failed to show external validity. It is important to note that clinical samples usually demonstrate much higher COPD prevalence, thereby spuriously increasing test performance. The majority of these studies also excluded never smokers, while in our scoring analysis, former, current as well as never-smokers were included. Furthermore, using pre-BD rather than post-BD spirometry falls short of current recommendations of COPD guidelines.

The primary aim of the Salzburg BOLD study and the primary care study was to estimate the population prevalence of COPD. We then used these data for the development of the SCSQ and possibly introduced a spectrum bias by not excluding subjects with a reported COPD diagnosis. However, as previously shown, as much as 50% of the subjects reporting a physician’s COPD diagnosis were false positive and failed to demonstrate airflow limitation.^42^

We decided to use the FEV_1_/FVC < LLN rather than the “fixed ratio” (FEV_1_/FVC < 0.7) to define airflow limitation. Since the FEV_1_/FVC ratio declines with age, using the fixed ratio of 0.70 will produce a greater number of false-positive results with increasing age, and the proportion of overdiagnosed COPD will be greatest in older populations.^43,44^

Verifying our statistical model by randomly splitting up the dataset (into a development and a validation sample) could have slightly overestimated sensitivity and specificity. However, when re-calculating these estimators without data splitting similar results were seen (data not shown).

Although the AUC did not demonstrate a meaningful cut-off point, we choose the ≥2 cutoff point, which we thought clinically useful and indicating reasonable sensitivity, specificity and NPV. Other COPD screening questionnaires showed better results regarding their discriminative power.^18,20–24^ This can be attributed to the different prevalence of symptoms and disease severity in varying settings, rather than the precision of the questionnaire. When using clinical symptoms to diagnose airways obstruction, higher specificity is observed in the general population, but higher sensitivity in the hospital setting.^45^ Therefore, a questionnaire should not be used straightforwardly in a different setting, unless its validity has been proven in this setting.

It is one of the strengths of the SCSQ that it was derived from a population-based study and therefore, subjects were not pre-selected neither with regard to smoking nor with regard to symptoms. Another strength is the use of PBD spirometry to define airway obstruction for COPD. The SCSQ was developed using the Austrian BOLD-data, and was externally validated in a primary care sample from the same population.^5,33^ The SCSQ will help to identify subjects for spirometry to detect airflow limitation, and thereby increase the efficiency of screening spirometry.

### Implications for future research, policy and practice

Several recommendations for the early detection of airflow limitation have been established. The National Lung Health Education Program^6^ advocated the use of office spirometry by primary-care for patients with cough, sputum production, or dyspnea, and/or a history of exposure to risk factors for COPD. On the other hand, the American College of Physicians and the U.S Preventive Services Task Force took a much more restrictive standpoint on the use of spirometry for COPD detection.^14^ Recent studies suggest that questionnaire-based screening can reduce the number of undiagnosed COPD, and is a feasible and effective way for preselecting patients for diagnostic spirometry.^19,46^ Other effective approaches for early COPD detection could be the use of handheld flow meters (e.g., Piko-6^®^) or combined risk prediction models.^47^ Such studies were successfully completed to prescreen for COPD in the primary care setting and in pharmacies.^19,47–50^

Early detection of COPD and successful stop smoking intervention will reduce future loss of lung function, and thereby also prevent the impeding deterioration of quality of life. As health resources are limited, spirometry should be targeted towards patients with either symptoms or the presence of risk for COPD. Furthermore, the beneficial effects of treatment of airways obstruction have only been shown in subjects with respiratory symptoms, and future studies are needed to evaluate the impact of early diagnosis on COPD management and quality of life.

## Conclusions

Altogether, primary and secondary prevention of COPD remains a challenge for already strained health care systems. Therefore, cost-effective ways to use these resources have to be sought, like pre-screening subjects for confirmatory spirometry. The SCSQ is a simple and readily applicable screening questionnaire for COPD and could be used to efficiently preselect for spirometry referral. Given its simplicity it could also be used for COPD detection and awareness campaigns in social media networks.

## Methods

### Population-based development sample and GP-based validation sample

In 2005, a gender-stratified random sample of 2200 adults aged >40 years was surveyed in Salzburg, Austria within the framework of the international BOLD-study. The validation sample was a random sample of patients from primary care offices of the same area^33^ and the BOLD protocol for PBD spirometry and questionnaire was applied.^5^ In both studies, exclusion criteria were used to guarantee the safety of spirometry testing. Participants were excluded from spirometry if they had one or more of the following conditions: recent chest or abdominal surgery, heart attack, detached retina, eye surgery in the past three months, hospitalization for any other heart problem in the last month, last trimester of pregnancy, resting pulse of greater than 120 beats per minute or current medication for tuberculosis. The local Ethics Committee of Salzburg approved both studies and all participants gave written informed consent.

### Study measures

Spirometry was done according to ATS/ERS recommendations^34^ by trained and certified technicians. A detailed description of study measures and methods has been described elsewhere.^3,35^

Non-reversible airflow limitation was defined as the FEV_1_/FVC ratio below the fifth percentile of the predicted value among a healthy never-smoking population (FEV_1_/FVC < LLN). The NHANES III reference equations were used to calculate predicted values and lower limits of normal^36^ Pack-years of cigarette smoking was defined as the average number of cigarettes smoked per day, divided by 20 (i.e., packs/day), times the duration of smoking in years. “Never smokers” smoked less than 20 packs of cigarettes their whole life, or smoked less than one cigarette per day over a period of one year.

### Data analysis

The screening questionnaire was developed in several stages. Firstly, we chose those variables of the BOLD-questionnaire that are either risk factors or symptoms indicating the presence of COPD (Table 4). All variables shown in Table 4 were used for multivariate analysis.

**Table 4:** Characteristics of subjects in the population-based BOLD-sample and the association with post-bronchodilator (PBD1) airflow limitation (*n* = 1258)

	PBD FEV_1_/FVC≥LLN	PBD FEV_1_/FVC<LLN
	(*n* = 1059)	(*n* = 199)
Age in years (mean, SE)	57.1 (0.34)	60.8 (0.89)
Male sex	597 (56.4%)	88 (44.2%)
Years of education > 12	168 (15.9%)	19 (9.6%)
Risk-Factors		
Smoking status		
Never smoker	532 (50.2%)	63 (31.7%)
Former smoker	355 (33.5%)	66 (33.2%)
Current smoker	172 (16.2%)	70 (35.2%)
Age when starting smoking (mean, SE)	18.6 (0.20)	20.0 (0.62)
Pack-years smoking >20	253 (48.0%)	82 (60.3%)
Worked in a dusty job	285 (26.9%)	61 (30.7%)
Farming	234 (22.1%)	54 (27.1%)
Passive smoking at home	225 (21.3%)	53 (26.6%)
Symptoms		
Wheezing or whistling at any time in the last 12 months	109 (10.3%)	56 (28.1%)
Cough without a cold	159 (15.0%)	64 (32.2%)
Phlegm	230 (21.7%)	82 (41.2%)
Shortness of breath interfering with daily activity	86 (8.1%)	38 (19.1%)
Own view about health “excellent”	135 (12.8%)	10 (5.0%)
Impact		
Breathing problems interfered with activity or caused to miss work	13 (1.2%)	9 (4.5%)
Health limits in climbing several flights of stairs	199 (18.8%)	79 (39.7%)

*PBD* Post-Bronchodilator, PBD *FEV1/FVC* ≥*LLN* post-bronchodilator FEV1/FVC ratio below the lower limit of normal (LLN), *SE* standard error

Development and testing of the COPD risk questionnaire was performed by a hold-out-method: We split the study sample (*n* = 1258) into two randomly assigned subsamples: a training subsample (*n* = 800) was used to create the risk-score, and a testing subsample was used to test it (*n* = 458). By using two sub-samples, the potential bias related to in-sample predictions was avoided.

The scoring model, based on the multivariate logistic regression, was calculated for the training subsample and tested on the testing subsample. Item reduction was performed by stepwise forward selection to receive the variables with the highest impact. The stepwise selection process consists of a series of alternating forward selection and backward elimination steps. The former adds variables to the model (applying Score test statistics), while the latter removes variables from the model (applying Wald test statistics). Firstly, a minimal model which did not contain any explaining variables was chosen. Secondly, items which showed a significant influence (forward selection) were included step by step.

The scores for each question were derived from the coefficients of the logistic regression model. To keep the calculation of the risk-score simple, coefficients were multiplied by two and rounded to the nearest integer. A summary score was calculated ranging from 0 to 9 points, indicating no/low risk (0 points) to high risk (9 points) for COPD.

In order to classify the risk of PBD FEV_1_/FVC < LLN, a cut-off-value was defined using the training-subsample. This cutoff-value was evaluated by applying the risk-score to the test subsample. Sensitivity, specificity, the negative predictive value (NPV), the positive predictive value (PPV), and the number needed to screen (NNS) were calculated. For the calculation of the NNS the prevalence of the BOLD sample was used. The area under the receiver operating characteristic (AUC) shows sensitivity and specificity associated with different cut-off-points. This COPD questionnaire derived from the BOLD sample was then validated in a patient sample from GP offices.^33^
